# Cognition and Aβ deposition predict grocery shopping performance in older adults

**DOI:** 10.1002/alz70857_103090

**Published:** 2025-12-25

**Authors:** Christopher A. Phillips, Cory A. Alcon, Sam N. Lockhart, Lisa A. Zukowski

**Affiliations:** ^1^ High Point University, High Point, NC, USA; ^2^ Wake Forest University School of Medicine, Winston‐Salem, NC, USA; ^3^ Perceptive Inc., Burlington, MA, USA

## Abstract

**Background:**

Better understanding how AD biomarkers (e.g., brain Aβ) and cognitive reserve predict performance on cognitively‐challenging tasks, like grocery shopping, in young (YA) and older adults (OA), could provide opportunities for earlier intervention. Therefore, this study aims to identify if demographics, social factors, cognitive performances, and Aβ deposition predict grocery shopping performance in YA and OA.

**Method:**

Fifty‐four OA (78±5 years) and 28 YA (31±3 years) without cognitive impairment from BNET [NCT03430427] performed four grocery shopping trials while wearing eye‐tracking glasses. Best and worst performances were analyzed, based on number of grocery note fixations and percentage of time fixating on correct shelving unit, brand, and shelf. Demographics (years of education, age), social factors (number in household), cognitive performances (global cognition [MoCA], information processing speed [Coding], set‐shifting ability [SSI], inhibition control ability [ICI]), and Aβ (PiB) PET Centiloids (OA only) were assessed as predictors. Individual simple and multiple linear regressions were performed for each group‐performance (YA‐best, YA‐worst, OA‐best, OA‐worst) to assess relationships between significant predictor variables, from correlation analyses, and respective grocery shopping measures.

**Result:**

Within YA‐best, worse MoCA and ICI scores predicted more frequent grocery note fixations (R^2^ = 0.267, *p* = 0.020). Less education predicted larger time percentages fixating on the correct shelving unit (R^2^ = 0.181, *p* = 0.024) and, in conjunction with better SSI scores, on the correct brand (R^2^ = 0.355, *p* = 0.004). Within OA, increasing age alone and in conjunction with greater Aβ and better Coding scores predicted more frequent grocery note fixations, respectively (OA‐worst: R^2^ = 0.113, *p* = 0.013; OA‐best: R^2^ = 0.297, *p* <0.001). During OA‐best, better Coding scores predicted a smaller time percentage fixating on the correct shelf (R^2^ = 0.145, *p* = 0.005). Finally, during OA‐worst, better MoCA scores predicted a larger time percentage fixating on the correct shelving unit (R^2^ = 0.139, *p* = 0.005), and, in conjunction with lower Aβ, larger time percentages fixating on the correct brand (R^2^ = 0.139, *p* = 0.022) and shelf (R^2^ = 0.259, *p* = 0.001).

**Conclusion:**

While relationships between predictors and grocery shopping performance differed between YA and OA, better cognition predicted better grocery shopping performance (less frequent note fixations and larger percentages of time fixating on relevant grocery regions) in both. Greater brain Aβ predicted less time spent fixating on relevant grocery regions.

